# Immune Responses Elicited in Tertiary Lymphoid Tissues Display Distinctive Features

**DOI:** 10.1371/journal.pone.0011398

**Published:** 2010-06-30

**Authors:** Olivier Thaunat, Stéphanie Graff-Dubois, Sophie Brouard, Chantal Gautreau, Aditi Varthaman, Nicole Fabien, Anne-Christine Field, Liliane Louedec, Jianping Dai, Etienne Joly, Emmanuel Morelon, Jean-Paul Soulillou, Jean-Baptiste Michel, Antonino Nicoletti

**Affiliations:** 1 Université Lyon 1, Villeurbanne, France; 2 Unité 851, Institut national de la Santé et de la Recherche Médicale, Lyon, France; 3 Service de Transplantation Rénale et d'Immunologie Clinique, Hôpital Edouard Herriot, Lyon, France; 4 Unité 698, Institut national de la Santé et de la Recherche Médicale, Hôpital Xavier Bichat, Paris, France; 5 Unité Mixte de Recherche en Santé 643, Institut de Transplantation et de Recherche en Transplantation, Hôpital Hôtel Dieu, Nantes, France; 6 Laboratoire Régional d'Histocompatibilité IR7, Assistance publique-Hôpitaux de Paris, Hôpital Saint Louis, Paris, France; 7 Laboratoire d'immunologie, Hospices Civils de Lyon, Centre Hospitalier Lyon-Sud, Pierre-Benite, France; 8 Molecular Immunology Unit, Institute of Child Health, University College London, London, United Kingdom; 9 Unité Mixte de Recherche 5089, Institut de pharmacologie et de biologie structurale, Toulouse, France; 10 Université Denis Diderot - Paris VII, Paris, France; Cairo University, Egypt

## Abstract

During chronic inflammation, immune effectors progressively organize themselves into a functional tertiary lymphoid tissue (TLT) within the targeted organ. TLT has been observed in a wide range of chronic inflammatory conditions but its pathophysiological significance remains unknown. We used the rat aortic interposition model in which a TLT has been evidenced in the adventitia of chronically rejected allografts one month after transplantation. The immune responses elicited in adventitial TLT and those taking place in spleen and draining lymph nodes (LN) were compared in terms of antibody production, T cell activation and repertoire perturbations. The anti-MHC humoral response was more intense and more diverse in TLT. This difference was associated with an increased percentage of activated CD4+ T cells and a symmetric reduction of regulatory T cell subsets. Moreover, TCR repertoire perturbations in TLT were not only increased and different from the common pattern observed in spleen and LN but also “stochastic,” since each recipient displayed a specific pattern. We propose that the abnormal activation of CD4+ T cells promotes the development of an exaggerated pathogenic immune humoral response in TLT. Preliminary findings suggest that this phenomenon i) is due to a defective immune regulation in this non-professional inflammatory-induced lymphoid tissue, and ii) also occurs in human chronically rejected grafts.

## Introduction

The progression towards chronic inflammation is characterized by a gradual shift in the type of immune effectors present at the site of inflammation i.e. an enrichment in cells from the adaptive immune system [1]. Besides this change in the composition of the inflammatory infiltrate, the organization of infiltrated cells is also modified. Indeed, it has long been observed that the inflammatory cells can organize themselves into structures displaying the same microarchitecture as secondary lymphoid organs [2]. The process by which a highly organized tertiary lymphoid tissue (TLT) arise *de novo* during chronic inflammation has been referred to as lymphoid neogenesis [3].

Immune response elicited in TLT develops in a microenvironment that differs from canonical secondary lymphoid organs because: i) surrounding inflammatory cells produce huge amounts of cytokines [4] and growth factors [5], ii) injured tissue constantly releases neoantigens, iii) defective lymphatic drainage traps neoantigens and immune effectors [6], and iv) absence of prepositioned regulatory subsets in TLT. We therefore hypothesized that immune response elicited in TLT could display distinctive features.

Chronic rejection, a prototypical chronic inflammatory disease, is an optimal situation to address this question since tertiary lymphoid tissues have systematically been detected in chronically rejected grafts [7], [8], [9], and the antigens targeted by the immune system are known (recipient-mismatched HLA antigens of the transplanted tissues). The aortic orthotopic transplantation between histoincompatible rat strains is a reliable model for chronic rejection [10] and a previous study has documented the development of TLT in the adventitia of chronically rejected allogenic aorta one month post-transplantation [9].

We therefore compared the characteristics of the immune responses elicited in the spleen, the draining lymph node, and the adventitial TLT during the chronic rejection of rat aortic allografts.

## Results

### TLT develops in the adventitia of chronically rejected aortic allograft

**Figure 1:**
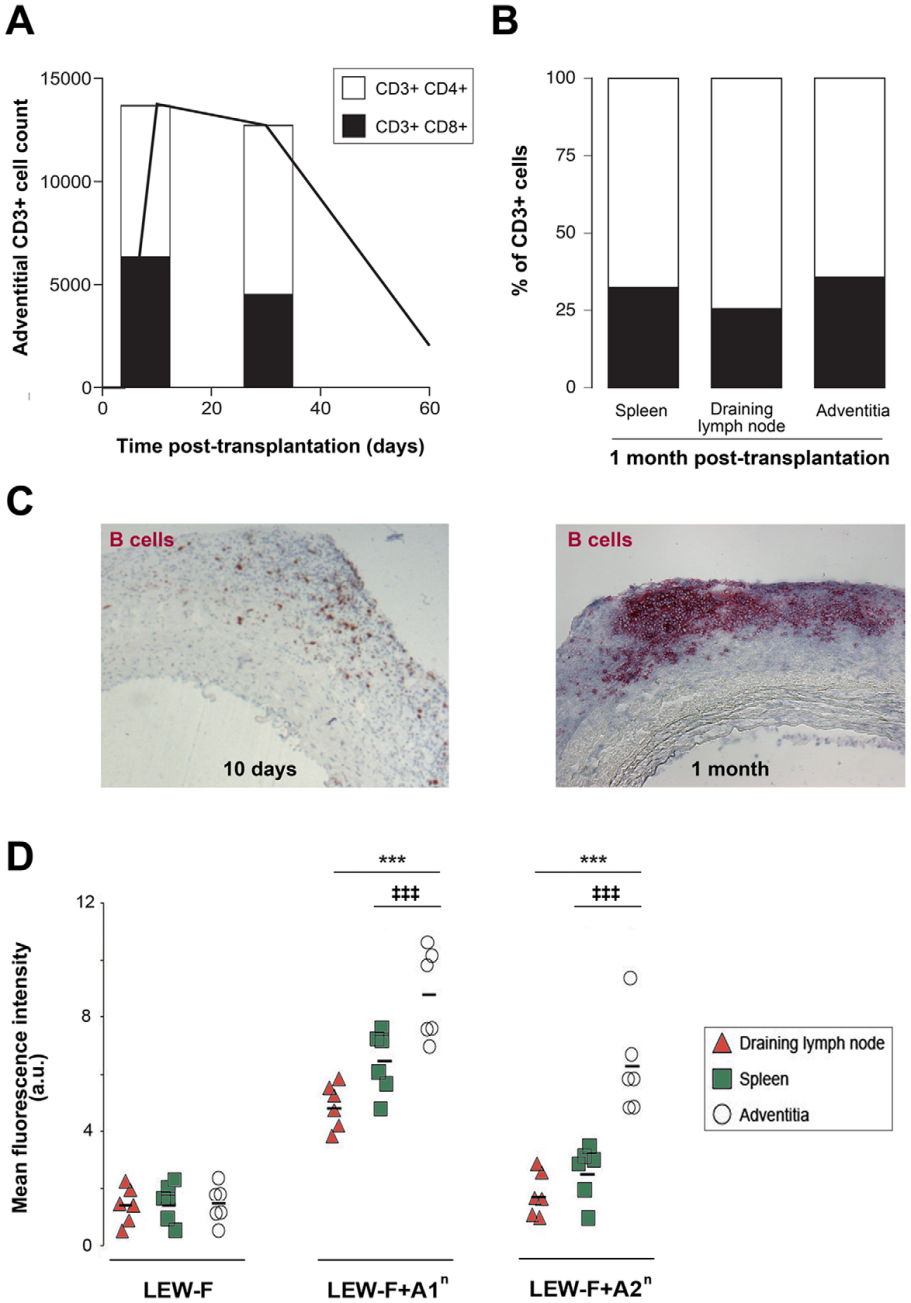
Exaggerated humoral response develops in TLT. **A.** The number of CD3+ cells (black line) and the proportions of CD4+ and CD8+ subsets (histogram) infiltrating the adventitia of rat aortic allografts were measured 10 days, 1 month and 2 months post-transplantation. The low number of cells at 2 months precluded the analysis of T lymphocyte phenotype at this time point. **B.** The distribution of CD4+ and CD8+ subpopulations among CD3+ cells was similar in the three lymphoid organs one month post-transplantation. **C.** Transversal sections of aortic allografts were stained with an anti-B lymphocyte monoclonal antibody. The scattered pattern of infiltration observed in the adventitia at 10 days **(left panel)** was replaced by an organized tertiary lymphoid tissue at 1 month **(right panel)**. **D.** The amount and the specificity of the antibodies present in the supernatants from tissue cultures of adventitia of chronically rejected aortic allograft, draining lymph nodes, and spleen was analyzed by flow cytometry using a Lewis (recipient) fibroblast cell line (LEW-F; negative control) and the same cell line transfected with the RT1.A1^n^ (LEW-F+A1^n^) or the RT1.A2^n^ (LEW-F+A2^n^) donor MHC I molecules. Cells were incubated with 100 µl of each supernatant. The binding of antibodies on the cell surface was determined with a FITC-conjugated anti-rat Ig κ light chain antibody by measuring the mean fluorescence intensity in a flow cytometer. Cell suspensions from the spleen, the draining lymph nodes and the adventitia of chronically rejected aortic graft were obtained from 10 recipient rats 1 month post-transplantation and analyzed by flow cytometry. Adventitia *vs* spleen: ‡‡‡ p<0.001; adventitia *vs* draining lymph node: *** p<0.001.

Kinetic analysis of aortic allograft rejection corroborated our previous observations [9], [11]: infiltration of the adventitia by recipient's lymphocytes began 5 days post transplantation, increased rapidly to peak at 10–15 days, remained stable 2–4 weeks and decreased thereafter, leaving an acellular fibrous scar 2 months post-transplantation **(** **Figure 1A** **)**. Of note, because the number of cells in the adventitia was very low at this time point, no reliable analysis of adventitial infiltrate could be performed.

T lymphocytes were the main cell population infiltrating the adventitia. Initially, T cell infiltrate was made of similar proportions of CD8+ and CD4+ T cells but the percentage of the latter tended to increase and helper T cells were the dominant subset 1 month post-transplantation **(** **Figure 1A** **)**. Of note, one month post-transplantation, the distribution of the CD4+ and CD8+ subpopulations among the CD3+ T lymphocytes was similar in spleen, draining lymph node and adventitial TLT **(** **Figure 1B** **)**.

In line with what we have previously reported [9], the scattered immune cells infiltrating the adventitia at the beginning of aortic allograft rejection **(** **Figure 1 C** **, left panel)** progressively structured themselves into an ectopic tertiary lymphoid tissue (TLT) typified by its organized microarchitecture observed 1 month post-transplantation **(** **Figure 1 C** **, right panel)**.

### Anti-MHC humoral response is more intense and more diverse in TLT than in canonical secondary lymphoid organs

Chronically rejected aortic grafts, recipient spleen and draining lymph nodes, were harvested 1 month post-transplantation and tissue-cultured so as to collect immunoglobulins produced within these tissues. The amount of anti-donor alloantibodies in tissue-culture supernatants was quantified by flow cytometry. The supernatants were tested on recipient fibroblasts, or on the same cell line transfected with the RT1.A1^n^ or the RT1.A2^n^ donor MHC molecules **(** **Figure 1D** **)**. We found that TLT produced 1.5 to 2 fold more alloantibodies than spleen and lymph nodes. Furthermore, the humoral response elicited in TLT appeared more diverse since reactivity against RT1.A2^n^, known to display a lower allogenic potential than RT1.A1^n^ [12], was detected only in the supernatant of aortic allograft tissue cultures.

### Over-activation of CD4+ T lymphocytes in TLT

Because the development of humoral responses against donor's MHC molecules is known to be dependant upon the help of CD4+ T cells to B cells [13], [14], [15], we focused the analysis on T helper subset.

**Figure 2:**
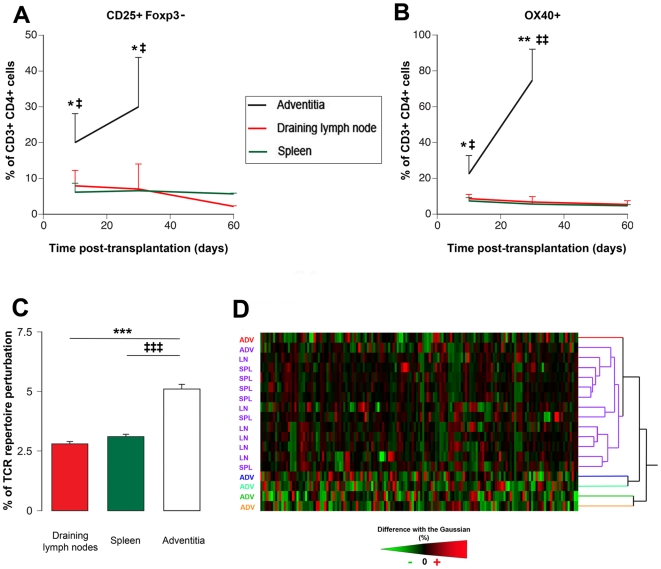
Characteristics of the T helper response in the various lymphoid organs participating in chronic rejection. **A. B.** The percentage of activated T lymphocytes was evaluated by: **A.** the expression of the α chain of the IL2 receptor (CD25), and **B.** the costimulatory molecule OX40 (CD134). Adventitia *vs* spleen: ‡ p<0.05, ‡‡ p<0.01; adventitia *vs* draining lymph node: * p<0.05, ** p<0.01. **C. D.** The TCR repertoire perturbations induced by chronic rejection in the spleen, the draining lymph nodes and the adventitial TLT were analyzed using the immunoscope method. Immunoscope divides T cell population into 180 “groups” defined upon the rearranged variable ß (Vß) gene segment used, and the length of the TCR CDR-3. In baseline conditions, the distribution profile of CDR3 lengths for each Vß family displays a Gaussian distribution. An increase in the height of a size peak signals an oligoclonal or a monoclonal expansion of this group that can be reliably quantified by the percentage of difference between the measured value the expected normal value. **C.** The mean percentage of perturbation for the 180 groups is shown for each lymphoid organ. Adventitia *vs* spleen: ‡‡‡ p<0.001; adventitia *vs* draining lymph node: *** p<0.001. **D.** The set of data was computed to group samples according to their pattern of TCR repertoire perturbation (Ward hierarchical clustering). Individual samples are listed in raw (ADV: adventitia; LN: draining lymph node; SPL: spleen), the 180 groups constituting the T cell population are in column. Perturbations are encoded from light green to bright red. On the right of the color map, a dendrogram list each observation, and shows which cluster it is in and when it entered its cluster.

We found that the percentage of activated CD4+ T cells, i.e. Foxp3− CD25+, was markedly increased in TLT as compared with the 2 canonical lymphoid tissues. This difference already significant 10 days post transplantation tended to increase at 1 month **(** **Figure 2A** **)**.

Strikingly, this over-activation coincided with the over-expression of OX40 **(** **Figure 2B** **)**, a costimulatory molecule from the TNF super-family known to play a crucial role in T cell-dependent help for humoral immune responses [16], [17].

### Stochastic bias in the TCR repertoire of TLT

Although providing a plausible explanation for the increased amount of alloantibody produced in adventitial TLT, the mere over-activation of CD4+ T cells cannot explain the more diverse repertoire of intragraft humoral response. In an attempt to understand the mechanisms underlying this distinctive feature of TLT humoral response, an analysis of the TCR repertoire was performed using an RT-PCR based approach named “Immunoscope” [18]. This method subdivides the bulk T cell population into 180 “groups” defined upon i) the rearranged variable ß (Vß) gene segment used, and ii) the length of the T cell receptor (TCR) third complementarity determining region (CDR3). The distribution profile of CDR3 lengths for each of the 20 Vß families is typically represented with 7–11 peaks each separated by 3 nuclear templates. Each peak represents a group of T cell clones using the same Vß gene segment and sharing the same CDR3 length.

In baseline conditions, the distribution profile of CDR3 lengths for each Vß family displays a Gaussian distribution [19]. Oligoclonal or monoclonal expansions are therefore reliably quantified by the percentage of difference between the observed peak size distribution value and the expected normal value [20].

During chronic rejection, the alloimmune responses taking place in the spleen and the draining LN induced a similar level of perturbation (∼3%) of the TCR repertoire **(** **Figure 2C** **)**. Interestingly, we found that the TCR repertoire in intragraft TLT was at least two fold more perturbed **(** **Figure 2C** **)**.

The data set was subsequently processed using the Ward's method for hierarchical clusterization allowing grouping the samples sharing similar TCR repertoire perturbations **(** **Figure 2D** **)**. We found that all the “canonical” lymphoid organs were in the same cluster (violet cluster in **Figure 2D**), with a strong tendency for spleens and lymph nodes to form sub-clusters according to the nature of the tissue. Surprisingly, we found that none but one adventitial TLT was distributed in the violet cluster. More interestingly the adventitial TLT, were not assembled in a cluster but rather split apart (as assessed by the hierarchical tree in **Figure 2D**), suggesting that each of the adventitial TLT displayed original, “stochastic”, perturbations of the TCR repertoire.

### Clues for a defective immune regulation in TLT

**Figure 3:**
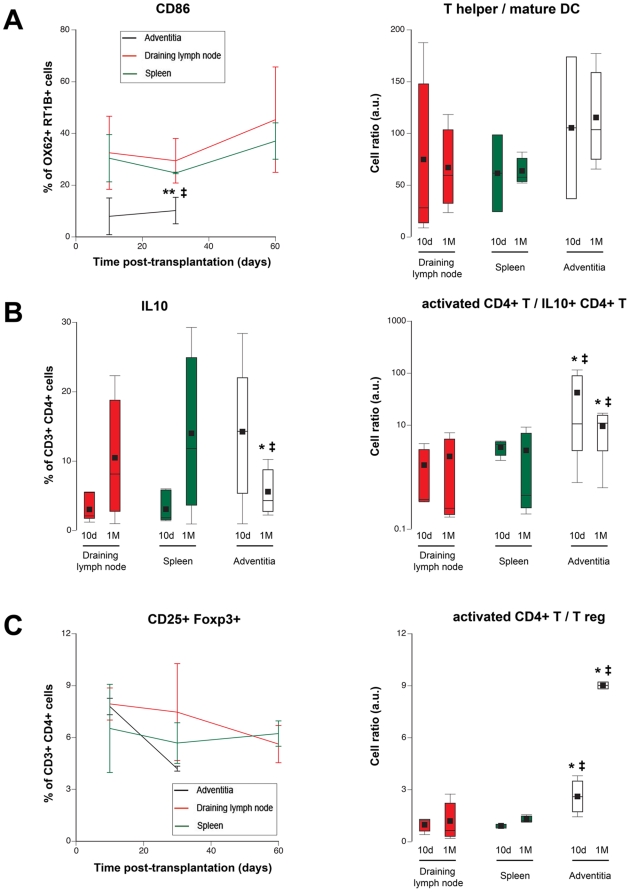
Clues for a defective immune regulation in TLT. **A.** The percentage of CD86-expressing mature dendritic cells (OX62+ MHC II+) was measured in the three lymphoid organs (left panel). The ratio: number of CD4+ T cells/number of mature dendritic cells was also calculated (right panel). Adventitia *vs* spleen: ‡ p<0.05; adventitia *vs* draining lymph node: ** p<0.01. **B.** The percentage of IL10-producing CD4+ T cells (Tr1) dropped in TLT between 10 days and 1 month post-transplantation, an evolution inverted as compared with spleen and draining lymph node (left panel). The ratio: number of activated (CD25+ Foxp3−) CD4+ T cells/number of Tr1 cells was increased in TLT at each time points (right panel). Adventitia *vs* spleen: ‡ p<0.05; adventitia *vs* draining lymph node: * p<0.05. **C.** The percentage of regulatory T cells (CD4+ CD25+ Foxp3+) tended to decrease in TLT between 10 days and 1 month post-transplantation (left panel). The ratio: number of activated (CD25+ Foxp3−) CD4+ T cells/number of T reg was increased in TLT at each time points (right panel). Adventitia *vs* spleen: ‡ p<0.05; adventitia *vs* draining lymph node: * p<0.05.

Since the activation of CD4+ T cells is critically depend upon professional antigen presenting cells, the number and the proportion of mature dendritic cells (DC) were compared in adventitial TLT, draining lymph node and spleen. The percentage of CD86-expressing mature DC, was significantly reduced in TLT at every time points **(** **Figure 3A** **, left panel)**. Moreover, the ratio between the number of CD4+ T cells and mature DC was also increased in TLT **(** **Figure 3A** **, right panel)**, making increased priming by DC an unlikely sufficient explanation for the adventitial stochastic over-activation of CD4+ T cells.

Alternatively, CD4+ T cell over-activation in TLT could be the consequence of a defective immune regulation in this “non-canonical” lymphoid tissue. In accordance with this latter hypothesis, a drop in T cell subsets endowed with immune regulatory properties was observed in TLT 1 month post-transplantation. Indeed, while the proportion of IL10-producing CD4+ T cells, *i.e.* Tr1 cells [21], tended to be higher in TLT 10 days post-transplantation, this percentage declined at one month post-transplantation, an opposite evolution as compared as what was observed in canonical secondary lymphoid organs **(** **Figure 3B** **, left panel)**. Of note, when the ratio between activated CD4+ T cells and Tr1 cells were compared, they were always significantly higher in TLT **(** **Figure 3B** **, right panel)**. Furthermore, similar observations were also made for another major regulatory subset [22]: the CD4+ CD25+ Foxp3+ T cells **(** **Figure 3C** **)**.

### Clinical relevance of experimental findings

Our group has previously reported the development of functional TLT within chronically rejected human grafts [9], [23], [24]. However, paired analysis of the immune responses elicited in the spleen, the draining lymph node, and intragraft TLT is unachievable in the clinical setting. Taking advantage of the explantation of certain chronically rejected renal grafts, we performed tissue culture experiments and compared the repertoire of intragraft-produced anti-HLA alloantibodies with the repertoire of circulating anti-HLA alloantibodies (assuming that the latter reflects the repertoire of the humoral responses elicited in the spleen and the lymph nodes).

**Figure 4:**
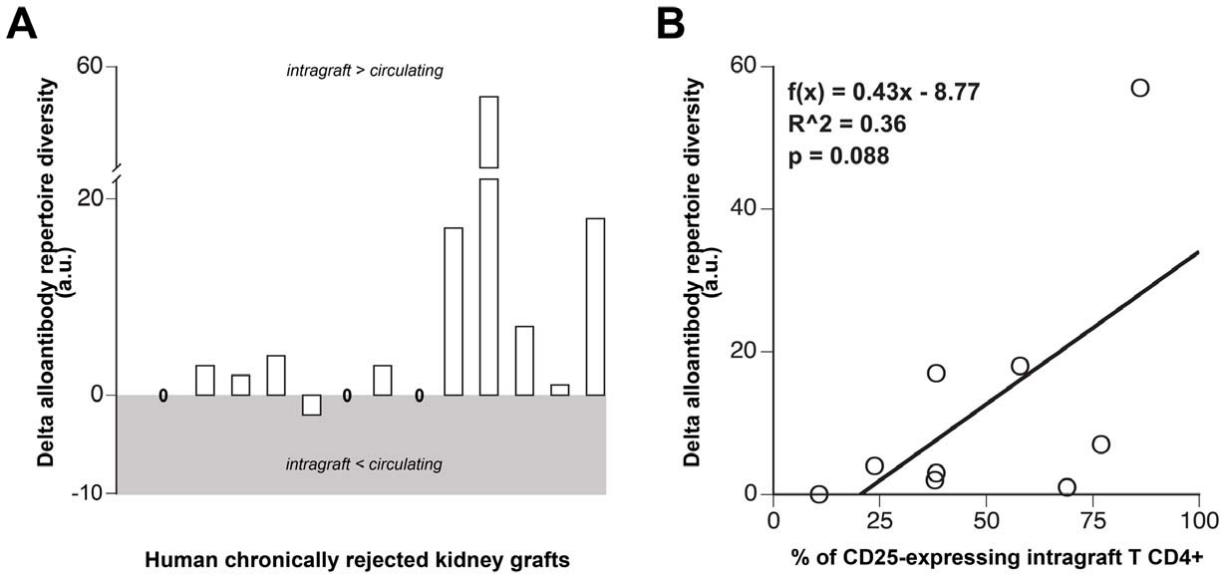
Analysis of chronically rejected human renal grafts. **A.** Fourteen human renal grafts, explanted for terminal chronic rejection, were analyzed. Anti-HLA antibodies were sought by luminex in tissue-culture supernatants and in the sera obtained immediately before detransplantation. Delta alloantibody repertoire diversity was calculated as the difference between the number of specificities identified in the tissue-culture supernatants and in the serum. No alloantibody was detected for 3 samples (0), among the remaining 11, the diversity of the repertoire of intragraft-produced alloantibodies was > to the diversity of circulating alloantibodies in 9 (82%). **B.** The percentage of activated (CD25+) CD4+ T cells infiltrating these 9 chronically rejected renal grafts was measured by flow cytometry. The linear regression model showed a trend for a positive correlation between the percentage of activated CD4+ T cells and the delta alloantibody repertoire diversity. The formula of the linear regression is provided, as well as R^2^: the coefficient of determination, and the p value of the regression.

Fourteen chronically rejected human renal grafts were analyzed **(** **Figure 4A** **)**. No anti-HLA antibody was detected, (neither in the circulation, nor in tissue culture supernatants) in 3 samples. In the remaining 11, the diversity of the repertoire of intragraft-produced alloantibodies was > to the diversity of circulating alloantibodies in 9 (82%). Strikingly, a consistent trend (p = 0.088) for a positive correlation between intragraft humoral response diversity and the percentage of CD25-expressing activated CD4+ T cells infiltrating these 9 grafts was observed **(** **Figure 4B** **)**.

## Discussion

In the present study we compared the immune responses elicited in intragraft TLT, spleen and draining lymph nodes during chronic rejection of a rat aortic allograft. We observed an increased production of alloantibodies in TLT as compared with canonical secondary lymphoid organs. Not only were the humoral alloimmune responses elicited in TLT quantitatively enhanced, but they also displayed a more diverse repertoire, a finding that we have validated in the clinical setting by the analysis of chronically rejected human kidney allografts.

Given the critical role of CD4+ T cells in the generation of the humoral alloimmune response [13], [14], [15], we hypothesized that the difference in the production of alloantibodies could reflect disparities between the T cell responses elicited in the various immune compartments. Accordingly, we observed a drastic increase in the percentage of activated CD4+ T cell in intragraft TLT, in both murine experimental model and human samples. The comparative analysis of the TCR repertoires of the three lymphoid tissues showed that during experimental chronic rejection, the TCR repertoire in intragraft TLT was at least two fold more perturbed than in canonical secondary lymphoid organs. At this stage, two hypotheses could be proposed to explain our results: i) an increased expansion of the alloreactive T cell clones in TLT but of the same specificities as those of the spleen and the draining LN, or ii) a broader activation of T lymphocytes in TLT resulting in the activation of additional T cell clones that were found resting in the draining LN and the spleen. To test these two hypotheses the ward's clustering method was applied to the dataset in order to group the samples together that shared similar TCR repertoire perturbation profiles. All the canonical secondary lymphoid organs were grouped in a single cluster. Surprisingly, none but one adventitial TLT was distributed in this cluster. Moreover, the adventitial TLT were not assembled in a cluster but rather split apart, suggesting that each of the adventitial TLT displayed original, “stochastic”, perturbations of the TCR repertoire. These results were unexpected since the TCR repertoire analysis was conducted at the same time point during the course of a chronic rejection in genetically identical couples of donors and recipients.

The activation of a CD4+ T lymphocyte requires the presentation of the adequate MHC class II-peptide complex (specifically recognized by the TCR of the T cell) by a mature recipient antigen presenting cells (APC) expressing costimulatory molecules [25]. The proportion of mature DC was not increased in TLT. However, the finding that the TCR perturbations are broad and stochastic suggests that the library of the complex MHC II-peptide presented by DC in TLT is more diverse. This is not surprising since i) TLT lay within the targeted tissues and all the neoantigens generated by the rejection process are therefore immediately accessible for the DC, and ii) chronically rejected organs feature a defective lymphatic drainage [6], [26] leading to the sequestration of the antigens and the APC at the rejection site.

A plausible explanation for the abnormal activation of CD4+ T cells in TLT could be relative to a defect in the mechanisms controlling the T cell response in this “non-professional” inflammatory-induced lymphoid tissue. One of such mechanism is dependent upon the regulatory subsets of CD4+ T cells [21], [22]. Interestingly, the amount of IL10-producing Tr1 cells, shown to be critical for the control of “determinant spreading“ during chronic immune responses [27], was found drastically reduced in TLT. The same observation was made for the T reg subset.

We conclude that during chronic rejection, the local alloimmune response elicited in intragraft TLT differs from the one taking place in the spleen and the draining lymph nodes. In particular, TLT CD4+ T cell activation i) is quantitatively increased, ii) is characterized by the activation of a broader range of T cell clones, iii) is stochastic, *i.e.* characterized by the absence of shared pattern of TCR perturbation in genetically identical couples of donors and recipients. This abnormal activation of CD4+ T lymphocytes correlates with a more intense and more diverse production of alloantibodies in TLT than in canonical secondary lymphoid organs. Our preliminary findings suggest that the oversized immune response in TLT is the consequence of a defect in the regulatory mechanisms.

## Materials and Methods

### Animals

Age-matched male Brown-Norway (BN; RT1^n^) and Lewis rats (LEW; RT1^l^) were obtained from Charles River (France). LEW rats were used as recipients and syngeneic donors, BN rats as allogeneic donors. All animal experimentation was undertaken in compliance with the European Community standards (authorization n° 75–214) and with the approval of the local Animal Experimentation Committee. Animals were kept under conventional conditions and fed a standard diet.

### Aorta transplantation

Rats were anesthetized with 50mg/Kg of pentobarbital injected intraperitoneally. Two animals were operated simultaneously, one as the donor of aortic graft and the other as the recipient, with the aid of an operating microscope. A 1 cm long segment of the donor abdominal aorta was excised, perfused with normal saline and small collateral arteries that originated from the graft were ligated. The donor aorta was transplanted in orthotopic position by end-to-end anastomosis in the recipient aorta below the renal arteries and above the iliac bifurcation. No immunosuppressive or anticoagulant treatment was used. One month post-transplantation, aortic grafts were removed from the Lewis recipients under anesthesia and perfused with saline. A total of fifty aortic allotransplantations and six aortic isotransplantations were performed.

### Immunohistological analysis

Ten micrometer-thick transversal cryosections of aortic allografts were air dried and fixed in acetone. Endogenous biotin and avidin were blocked (Biotin-avidin Block, Dako, France). B lymphocytes were stained using the mouse anti-pan rat B cell antibody (RLN-9D3; 1/100; Serotec). We used a biotin-conjugated horse anti-mouse secondary antibody (Vector Laboratories, USA). Immunohistochemical staining was revealed using alkaline phosphatase anti-alkaline phosphatase (APAAP) complexes.

Sections were counterstained with hematoxylin. Negative control slides were performed with the primary antibody omitted.

### Tissue cultures

Lymphoid tissue cultures were performed as previously described [9].

Briefly, draining lymph nodes, spleen, and adventitia were recovered from 6 Lewis recipients 1 month post-transplantation.

Tissues were weighted, washed three times and cultured 5 days in 2 ml sterile X-VIVO 15 serum-free medium (Cambrex, Walkersville,MD) at 37°C.

### Isolation of adventitial cells

Cells within the adventitia of the graft were isolated by microdissection, digestion in a collagenase I solution (Gibco/Invitrogen, France), and filtration through 100µm nylon meshes as previously described [9].

### Flow Cytometry

#### Antibodies

The following ant-rat antibodies were used: anti-CD3 (1F4), anti-CD4 (OX35), anti-CD25 (OX-39), anti-CD86 (24F), anti-CD103 (OX-62; dendritic cells), anti-CD134 (OX-40; Serotec), anti-Foxp3 (FJK-16s; eBioscience), anti-RT1B (OX-6; MHC class II molecule), and anti-TCRαβ (R73; Serotec). Unless indicated otherwise, reagents were from BD Biosciences.

#### Detection of cell surface antigens

Single cell suspensions of spleen cells, lymph nodes and adventitia from recipient rats were prepared. Cells (0.1 million) were stained with FITC-, PE-, Percp, PECy5-, or biotin-conjugated mAbs. Biotinylated mAbs were revealed with streptavidin-PE or -APCCy7 (BD Biosciences, France).

Absolute number of each cell population was determined by addition of Flow-count™ fluorospheres from Beckman Coulter just before data acquisition on a LSRII using the DIVA Software (BD Biosciences).

#### Quantification of T regulatory cells

Foxp3 staining was performed, after staining of CD3, CD4 and CD25 surface antigens, using the Foxp3 buffer set from eBioscience according to the manufacturer instructions.

#### Detection of intracellular IL10

The quantification of IL10-producing T cells was performed by combined surface and intracellular staining with mAbs and subsequent flow cytometric analysis. Adventitial lymphocytes were stimulated with PMA (50 ng/ml; Sigma-Aldrich, France) and ionomycin (1 µg/ml; Calbiochem, CA) for 6 hours and cytokine secretion inhibited by treatment with 10 µg/ml brefeldin A (Alexis Biochemical, Switzerland) the last 2 hours of incubation. Stimulated cells were washed and stained with anti-TCRαβ and anti-CD4. Labeled cells were fixed and permeabilized with a 0.1% saponin solution (Sigma-Aldrich). Intracellular staining was performed with a PE-conjugated anti-rat IL10 antibody (A5-4; BD Biosciences). Cells were washed twice in a 0.1% saponin solution and re-suspended in PBS for flow cytometry analysis.

#### Analysis of the specificity of alloantibodies

The analysis of the specificity of the alloantibodies present in the tissue culture supernatants was performed by flow cytometry as previously described [9]. Briefly, the tissue culture supernatants were tested on Lewis (recipient) fibroblasts (LEW-F), or on the same cell line transfected with the RT1.A1^n^ (LEW-F+A1^n^) or the RT1.A2^n^ (LEW-F+A2^n^) Brown-Norway (donor) MHC I molecules [12]. The binding of antibodies on the cell surface was then determined with an anti-rat Ig κ light-chain FITC-conjugated antibody (MARK 1).

### Analysis of the TCR repertoire

#### RNA extraction and cDNA synthesis

RNA was isolated from the spleen, the draining lymph nodes, and the microdissected adventitia from 6 BN aortic alllografts, using the guanidinium isothiocyanate procedure and purified on a cesium chloride gradient [28]. RNA (10 µg) was reverse transcribed using a cDNA synthesis kit (Roche, Indianapolis, IN) and was diluted to a final volume of 100 µl.

#### Immunoscope

cDNA was amplified by PCR using a Cß primer and one of the 20 Vß-specific primers [29].The amplifications were performed in a 9600 PerkinElmer Automate (PE Applied Biosystems, Foster City, CA). PCR amplification conditions were as previously described [30]. Each amplification product was used for an elongation reaction using a dye-labeled Cß primer [29], then heat-denatured, loaded onto a 6% acrylamide-8 M urea gel, and electrophoresed for 5 h using an Applied Biosystems 373A DNA sequencer (PerkinElmer).

Immunoscope software (Institut Pasteur, Paris, France) provides distribution profiles of CDR3 lengths, in amino acids, of the amplified and elongated products [18]. Each profile is composed of between 7 and 11 peaks, spaced by three nucleotides, corresponding to 7 to 11 possible lengths of the CDR3 region. A given length of the CDR3 is not necessarily associated with the same sequence, and the number of transcripts with a given length is proportional to the area under the peak [18].

In baseline conditions, the distribution profile of CDR3 lengths for each Vß family displays a Gaussian distribution [19]. Oligoclonal or monoclonal expansions are therefore reliably quantified by the percentage of difference between the observed peak size distribution value and the expected normal value, providing that the size of the T lymphocyte population analyzed is ≥1 10^6^ cells [20]. Indeed, when the number of T cells is below this threshold, artifactual biases related to “sample effect” can occur. The technique of enzymatic digestion allowed to harvest only a fraction of the cells infiltrating the adventitia: 2.87±1.86 10^6^ among which 35.04±18.47% were T cells.

It was therefore impossible to carry out the individual TCR repertoire analysis using sorted adventitial CD4+ and CD8+ T lymphocytes. To ensure that the T cell population analyzed was >1 10^6^ cells, the Immunoscope analysis was performed on the total adventitial tissue.

### Human study

#### Chronically rejected human allografts

Fourteen renal allografts, removed due to terminal chronic active rejection, were collected in 4 transplantation centers. The tissues were maintained in germ-free conditions at 4°C and were processed <24 hours after explantation.

All the patients gave informed consent for the use of the samples for research purposes.

#### Tissue cultures

Twenty-four randomly selected fragments (∼0.5 mm3) of the renal cortex of freshly explanted allografts were washed 3 times and cultured in a 24-well plate in 1ml RPMI 1640 medium (Cambrex) supplemented with 100U/ml penicillin/streptomycin and 25µg/ml Fungizone (Gibco). Culture supernatants were harvested after 5 days of culture and stored at −20°C until further analysis.

#### Luminex

Luminex assays were used to detect the presence of anti-HLA alloantibodies in the supernatants (LifeScreen®, Tepnel Lifecodes Corporation) and subsequently, to determine their specificity (LABScreen® single antigen HLA class I and class II detection tests, One Lambda).

#### Flow cytometry

Fresh explanted grafts were cut with a sterile razor blade into ∼0.125mm3 fragments which were incubated in a solution of 1 mg/ml collagenase A and 0.1 mg/ml DNAse I (Roche) for 1 hour at 37°C. Cell suspensions were passed through a 70 µm cell strainer, and mononuclear cells were separated over Ficoll-Paque Plus (Amersham).

Ten million cells were incubated with a cocktail of fluorescent monoclonal antibodies specific for the following human cell surface markers: CD3 (PE-Texas Red, clone 7D6), CD4 (Alexa Fluor 700, clone RPA-T4), CD8 (Pacific Blue, clone RPA-T8), CD25 (FITC, clone M-A251), All these antibodies were from BD Biosciences except the anti-CD3 from Caltag Laboratories.

More than 1.106 events in the lymphoid FSC/SSC gate were acquired on an LSRII flow cytometer and analyzed with DIVA software (BD Biosciences).

### Statistical analysis

Data were analyzed using the JMP 6.0 software (SAS Institute Inc., Cary, NC). Statistical significance of results was determined by analysis of variance with one-way ANOVA followed by Fischer's PLSD tests; p values of less than 0.05 were considered as statistically significant.
